# 长链非编码RNA在非小细胞肺癌EGFR-TKIs耐药中的研究进展

**DOI:** 10.3779/j.issn.1009-3419.2018.02.06

**Published:** 2018-02-20

**Authors:** 树斌 李, 鸿 于, 耿月 张

**Affiliations:** 1 102600 北京, 中国中医科学院广安门医院南区内科 Department of Internal Medicine, Southern Branch of Guang'anmen Hospital, China Academy of Chinese Medical Sciences, Beijing 102600, China; 2 130012 长春, 吉林省肿瘤医院吉林省肿瘤防治研究所细胞生物研究室 Cell Biology Laboratory, Jilin Province Institute of Cancer Prevention and Treatment, Jilin Cancer Hospital, Changchun 130012, China

**Keywords:** 肺肿瘤, EGFR-TKIs, 耐药, 长链非编码RNA, Lung neoplasms, Epidermal growth factor receptor-tyrosine kinase inhibitors, Resistance, Long noncoding RNA

## Abstract

携带表皮生长因子受体（epidermal growth factor receptor, *EGFR*）突变的非小细胞肺癌（non-small cell lung cancer, NSCLC）患者经EGFR酪氨酸激酶抑制剂（EGFR tyrosine kinase inhibitors, EGFR-TKIs），如吉非替尼长期治疗后，其中的绝大部分患者会最终发生获得性耐药，导致疾病进展。EGFR-TKIs耐药涉及多种机制。目前对长链非编码RNA（long non-coding RNA, lncRNA）在EGFR-TKIs耐药中的作用还知之甚少。本文旨在对lncRNAs在NSCLC EGFR-TKIs耐药中的研究进展进行综述。

## Abstract (en)

Most non-small cell lung cancer patients with active epidermal growth factor receptor (*EGFR*) mutation will eventually acquire drug resistant to EGFR tyrosine kinase inhibitors, such as gefitinib, resulting in disease progression, which involves a variety of complex mechanisms. Up to now, the molecular mechanisms of long non-coding RNAs mediated EGFR-TKIs resistance remains poorly understood. This review aims to outline the current state of information on lncRNAs and progress on its role in EGFR-TKIs resistance in non-small cell lung cancer.

肺癌是最常见的恶性肿瘤之一，也是世界范围内癌症死亡的主要原因^[1]^。近年来，虽然在临床和实验肿瘤学方面取得了可喜的进展，但肺癌患者的预后仍不理想，5年总生存率仅在15%左右。这主要由于很大一部分患者在被确诊时已经处于癌症晚期，错过了最佳治疗时期。

非小细胞肺癌（non-small cell lung cancer, NSCLC）约占所有肺癌病例的85%，包括两个主要的组织学亚型：腺癌（lung adenocarcinoma, LAD）和鳞状细胞癌（squamous cell carcinoma, SCC）。2004年在NSCLC患者中首次发现表皮生长因子受体（epidermal growth factor receptor, *EGFR*）突变。虽然大约有10%的患者表现出原发性耐药，但是大多数携带*EGFR*突变的患者对EGFR酪氨酸激酶抑制剂（EGFR tyrosine kinase inhibitors, EGFR-TKIs）如厄洛替尼（erlotinib）和吉非替尼（gefitinib）治疗敏感。EGFR-TKIs的应用极大的提高了NSCLC患者的临床疗效，然而那些对治疗敏感的患者在治疗10个月-16个月后会不可避免的获得抗药性，导致癌症进展。

一些EGFR-TKIs耐药的机制已经被阐明，如c-MET扩增、*PIK3CA*突变、*EGFR*“gatekeeper”突变（T790M）、*BRAF*突变、NF-κB活化和小细胞肺癌转化等，但是仍有大约30%的病例其EGFR-TKIs耐药的机制是不清楚的。最近，高通量基因组分析显示，在人类肿瘤组织样本和肿瘤细胞系中，长链非编码RNA（long non-coding RNAs, lncRNAs）对于肿瘤的发生和发展都具有突出的重要作用。越来越多的研究证据也表明lncRNAs参与NSCLC的发生和进展。因此，深入研究lncRNAs的功能为阐明NSCLC的生物学机制开辟了新的途径，也为开发更有效的治疗手段提供了新的可能性。目前虽然已有报道lncRNAs，如HOTAIR^[2]^、MEG3^[3]^、CCAT1^[4]^等在NSCLC化疗耐药中发挥作用。但是NSCLC EGFR-TKIs耐药中lncRNAs的表达模式和潜在的功能仍然是未知的。本文总结了lncRNAs在NSCLC EGFR-TKIs耐药中的研究进展。

## 1 LncRNAs的胞内定位和作用机制

LncRNAs的长度大于200 nt（nucleotides），缺乏或没有蛋白编码能力，可参与多种生物学过程，包括X染色质印迹、细胞分化、选择性剪接、细胞命运决定、免疫反应、肿瘤发生等^[5]^。目前已经被鉴定的lncRNAs超过30, 000条。但是仅有1%的lncRNAs的功能被研究。

LncRNAs的功能是由其在细胞内的定位决定的。多数核内lncRNAs指导染色质修饰，它们通过招募DNA甲基转移酶3和组蛋白修饰基因，如polycomb repressive complex PRC2和histone H3 lysine 9（H3K9）甲基转移酶发挥转录抑制作用。此外，lncRNAs的自身转录也可以负调节基因表达。根据距离其作用位点的远近，核内lncRNAs的作用方式可分为cis-和trans-acting两种。cis-acting lncRNAs主要调控它们的转录位点附近的基因表达，而trans-acting lncRNAs调节远处独立位点的基因表达。目前这两种lncRNAs的作用机制都尚未完全被阐明。一些可能的机制包括招募桥蛋白，形成RNA-DNA三联体，通过RNA结构识别DNA。核内的lncRNAs对基因位点也有直接的调节作用，它们可以作为诱饵隔离转录因子、变构调节调节蛋白、改变核域和染色体的三维结构^[6]^。

胞质中lncRNAs调节翻译过程和mRNA的稳定性^[6]^。它们通过碱基配对对翻译过程进行调控。它们的另一个特别的功能是作为竞争性内源性RNAs（competing endogenous RNAs, ceRNAs）结合并阻断特异性的miRNAs，作为“miRNA sponges”保护靶mRNAs不被抑制。这一功能与肿瘤发生、细胞分化、多能性等很多生物学过程有关。此外，细胞质中一些含有小的开放阅读框（open reading frames, ORFs）的lncRNAs是能够被翻译成有生物学活性的小肽的。

由于lncRNAs的非保守性，它们可在不同水平通过不同的机制调节基因表达。在转录水平，lncRNAs一方面可以抑制转录因子和启动子的结合，通过与RNA PII的相互作用调节基因转录。另一方面lncRNAs也可作为ceRNA调控基因转录。此外，lncRNAs还有转录因子共激活子的功能，可以与DNA形成三螺旋结构影响靶基因的转录。在转录后水平，lncRNAs调节pre-mRNAs的选择性剪接，通过与miRNAs相互作用影响mRNA的翻译。lncRNAs可与mRNA形成双链RNA，提高mRNA的稳定性，也可被切割成小的非编码RNA行使mRNA调节功能。在表观遗传调控水平，lncRNAs控制DNA甲基化（影响启动子CpG岛甲基化），调节组蛋白修饰（甲基化、乙酰化和泛素化）。lncRNAs还可与染色质修饰复合物结合调控染色质重塑、基因表达和肿瘤发生等^[7]^。

在肿瘤的发生发展过程中，lncRNAs既能发挥促进作用也能产生抑制作用。在p53、NF-κB、PI3K/AKT等一些肿瘤相关的重要信号通路中，lncRNAs可作为信号级联中的受体、蛋白激酶、转录因子等的连接支架，将多种信号分子连接起来进而调控信号级联反应过程。p53信号的下游事件中有大量的lncRNAs参与，一些lnRNAs能够自反馈调节p53表达和稳定性，例如p53在应答DNA损伤时诱导Linc-RoR表达。Linc-RoR启动子中又含有一些保守的p53结合位点可与p53相互作用阻断p53的翻译。NF-κB信号通路失常与炎症、耐药、肿瘤转移密切相关。NF-κB-互作lncRNA（NF-κB-interacting lncRNA, NKILA）在NF-κB信号通路中起支架作用。NF-κB上调NKILA表达。NKILA又与NF-κB复合物相互作用，阻止IKK引发的IκB磷酸化，抑制NF-κB活化。在PI3K/AKT信号通路中，lncRNAs同样通过靶向信号通路的不同成分发挥独特的作用。值得注意的是，NF-κB是AKT的效应器。因此，AKT相关的lncRNAs在某种程度上也会影响NF-κB的活性^[8]^。

## 2 LncRNAs与NSCLC

与基因一致，lncRNAs也可分为原癌lncRNAs和肿瘤抑制lncRNAs。在NSCLC中前者包括MALAT1（metastasis-associated lung adenocarcinoma transcript 1）^[9]^、CCAT2（colon cancer-associated transcript 2）、HOTAIR（HOX transcript antisense intergenic RNA）、IGFBP4-1（insulin-like growth factor binding protein 4-1）^[10]^、TFPI2AS1（TFPI2的antisense transcript）^[11]^、LINC00152^[12]^、BLACAT1（bladder cancer associated transcript 1）^[13]^、LINC00968^[14]^、FAM83H-AS1（FAM83H antisense RNA1）^[15]^等。后者有AK126698、MEG3（maternally expressed gene 3）、GAS6-AS1（growth-arrest-specific gene 6 antisense 1 RNA）、BANCR（BRAF activated non-coding RNA）、RP11-713B9.1（TSLC1的antisense transcript）^[16]^、GAS5（growth arrest-specific transcript 5）^[17, 18]^等。

### 2.1 原癌lncRNAs与NSCLC

CCAT2在NSCLC尤其是LAD中高表达^[19]^，推测CCAT2通过TCF7L2介导的转录上调MYC、miR-17-5p、miR-20a表达，增强Wnt信号活性，促进细胞迁移。HOTAIR通过招募PRC2（polycomb repressive complex 2）或重组染色质促进多种肿瘤进展。初步的研究^[2]^已经证明HOTAIR通过影响p21表达促进细胞凋亡和G_0_期/G_1_期阻滞，介导NSCLC患者对顺铂耐药。linc00673在NSCLC组织中表达上调并与淋巴结转移和TNM分期正相关^[20]^。其作用机制可能是linc00673通过与miR-150-5p相互作用间接作用于上皮间质转化（epithelial mesenchymal transition, EMT）关键基因ZEB1促进NSCLC进展^[21]^。lncRNA NEAT1（nuclear enriched abundant transcript 1）在NSCLC组织样本中表达显著上调并与患者的不良预后正相关^[22, 23]^。有报道miR-377-3p（MIMAT0000730）与NEAT1相互作用。与细胞周期调节有关的E2F3（E2F transcription factor 3）携带2个保守的miR-377-3p同源位点，可能是miR-377-3p的靶基因^[24]^。NEAT1通过调节miR-377-3p/E2F3轴在NSCLC进展中发挥重要作用。此外，Li等的研究表明，lncRNA-NEAT1与miR-181a-5p相互作用，miR-181a-5p的靶基因是*HMGB2*。在NSCLC中lncRNA-NEAT1与miR-181a-5p竞争性作用于HMGB2^[25]^。Wu等^[26]^发现lncRNA-NEAT1与hsa-mir-98-5p相互作用，而hsa-mir-98-5p的靶基因是*MAPK6*。他们证明NEAT1/has-mir-98-5p/MAPK6在NSCLC进展中同样发挥重要作用。对lncRNA-NEAT1的研究充分说明了lncRNAs的作用机制的复杂性，未来有太多的问题需要深入探讨。

### 2.2 肿瘤抑制lncRNAs与NSCLC

AK126698的表达水平在NSCLC组织中显著降低，并且与肿瘤大小和病理分期相关^[27]^。Axin1、β-catenin、c-myc和cyclin D1是Wnt/β-catenin信号通路的重要组成部分。过表达的AK126698使FZD8（Frizzled-8，一个Wnt/β-catenin信号通路的受体）和β-catenin表达下调，Axin1和E-cadherin表达上调，从而增强NSCLC患者对顺铂治疗的敏感性^[28]^。MEG3在多种肿瘤中表达下调，促进肿瘤进展。在NSCLC中MEG3低表达的患者总生存时间减少^[29]^，可能是由于MEG3低表达导致p53表达降低造成的。GAS6-AS1表达降低有助于NSCLC进展^[30]^，可作为NSCLC的预后标志。其机制可能涉及到*GAS6*基因。GAS6是Axl/Sky的一个配体。GAS6/Axl是多种肿瘤侵袭转移所必需的。BANCR在NSCLC患者肿瘤组织中表达降低并与远处转移和患者的总生存时间减少有关^[31]^，其机制可能涉及EMT。NSCLC肿瘤组织中lncRNA-TUG1（taurine-upregulated gene 1）表达显著降低并且与患者的性别、吸烟史、肿瘤分化程度相关^[32]^。*CELF1*（CUGBP and Elav-like family member 1）是受TUG1负调节的靶基因。TUG1 RNA通过结合CELF1启动子区的PRC2负调节CELF1表达在NSCLC进展中发挥作用。

### 2.3 生物信息学分析结果

2016年，Zequn等^[33]^分析了人类大细胞肺癌细胞系的lncRNA和mRNA表达谱，发现二者之间有313个LncRNAs和252个mRNAs差异表达。其中LncRNA TATDN1（Homo sapiens TatDDNase domain containing 1）可能通过Wnt/β-catenin和PI3K/Akt/mTOR信号通路靶向β-catenin和Ezrin在肿瘤发展、侵袭和转移中发挥作用。Xu等^[34]^检测分析了肺腺癌组织的lncRNA和mRNA表达谱。发现差异表达的lncRNAs共有2, 420条。作者最终确认了2个差异表达最显著的lncRNAs，分别是高表达的LOC100132354和低表达的RPLP0P2。很显然，针对这两个lncRNAs的深入研究将有助于阐明肺腺癌的发生发展机制。Zhang等^[35]^也检测分析了肺腺癌组织的LncRNA表达谱并从中选择了差异表达最显著的LncRNA ZXF1深入研究。结果表明，肺癌组织的LncRNA ZXF1表达水平显著升高，并且与肿瘤淋巴结转移、病理分期、不良预后相关。

## 3 LncRNAs与NSCLC EGFR-TKIs耐药

LncRNAs在靶向药物耐药中的作用在一些肿瘤的研究中已有报道。如肾癌的Sunitinib耐药^[36]^、肝癌的sorafenib耐药^[37]^。本文主要关注在lncRNAs在NSCLC EGFR-TKIs耐药中的研究进展。

### 3.1 利用生物信息学方法分析lncRNAs与NSCLC EGFR-TKIs耐药的关系

随着生物信息学技术的飞速发展，很多研究人员开始使用公共数据集来分析人类疾病的lncRNA表达谱。Ma等^[38]^从GEO（Gene Expression Omnibus）数据库中下载了3个数据集，分别是gefitinib耐药的GSE34228，erlotinib耐药的GSE38310，crizotinib或X-376耐药的GSE49508。他们随后分析了这3个数据集的lncRNA表达谱。GSE34228的数据分析显示114个lncRNAs表达失调，其中59个lncRNAs表达上调，55个lncRNAs表达下调。GSE38310的分析结果表明，在ER3细胞中有43个lncRNAs表达失调，另有45个lncRNAs表达失调在T15-2克隆中。有9个lncRNAs在2个组中均表达上调，13个lncRNAs表达下调。分析GSE49508的结果显示，在PF-1066耐药和X-376耐药的H3122细胞中分别有51个lncRNAs和107个lncRNAs表达失调。其中9个lncRNAs表达上调、18个lncRNAs表达下调。作者从这些表达失调的lncRNAs中选取了上调的CASC9和下调的EWAST1（LINC00227）作为研究对象，用*Pearson*相关系数计算与之共表达的蛋白编码基因（protein-coding genes, PCGs）。结果表明，CASC9共表达的PCGs主要与选择性剪接、蛋白-DNA相互作用、核小体组装、外泌体、甲基化等功能有关。LINC00277共表达的PCGs主要参与蛋白磷酸化调节、蛋白结合、免疫反应等。推测CASC9和LINC00277可能通过与这些PCGs相互作用调节EGFR-TKIs耐药。

差异表达的lncRNAs可以作为逆转EGFR-TKIs耐药的候选靶点。Cheng等^[39]^分析了EGFR-TKIs敏感的PC9和耐药的PC9/R肺癌细胞的lncRNA表达谱，发现二者之间共有22, 587个lncRNAs和17, 479个mRNAs差异表达。其中1, 731个lncRNAs和2, 349个mRNAs在PC9/R细胞中表达上调，2, 936个lncRNAs和1, 307个mRNAs在PC9/R细胞中表达下调。共有699个基因与细胞增殖和凋亡有关。GO分析结果表明，上调的转录本主要负责细胞代谢过程、细胞内组分、蛋白结合等。下调的转录本主要负责泌尿生殖系统发育、细胞外空间以及蛋白结合等。Pathway分析结果表明，上调的转录本主要涉及RNA运输、溶酶体、spliceosome等19条信号通路。下调的转录本主要涉及沙门氏菌感染、花生四烯酸代谢、legionellosis等17条信号通路。这其中与细胞增殖和凋亡相关的信号通路在EGFR-TKIs耐药中发挥重要作用。作者随后采用RT-qPCR方法验证了4个lncRNAs（H19、BC200、MALAT1、HOTAIR）的表达，以证明这些lncRNAs可能作为EGFR-TKIs治疗的潜在靶点。这项研究的完成给了我们很多启示。已知lncRNAs可以调节邻近的蛋白编码基因的表达。作者发现上调的lncRNA-BC087858位于一个与癌症发展相关的FOX转录因子家族成员FOXC1（forkhead box protein C1）附近。FOXC1可以通过抑制E-cadherin表达诱导EMT，促进细胞迁移和侵袭。FOXC1的表达也可以被EGF/ERK（epidermal growth factor/extracellular signal-related kinase）信号通路激发^[40]^。因此作者推测BC087858可能通过EMT在EGFR-TKIs耐药中发挥作用。又比如lncRNA-RP11-15H7.2位于CITED2附近，而CITED2是一个与癌症有关的转录调节子，被报道与顺铂耐药相关^[41]^。因此，lncRNA-RP11-15H7.2也可能在EGFR-TKIs耐药中发挥作用。

LncRNAs调节RNA的剪接、稳定、核质穿梭，因此蛋白编码RNAs和lncRNAs的共表达分析可以反映lncRNAs的功能。Xue等^[42]^利用GEO的数据集GSE49644和GSE60189构建了肺癌耐药的lncRNAs-mRNAs共表达功能性网络。他们首先分析了mRNA和lncRNA表达谱，筛选出差异表达显著的34个lncRNAs和103个mRNAs。其中10个lncRNAs和49个mRNAs表达下调，24个lncRNAs和54个mRNAs表达上调。GO分析显示差异表达的mRNAs主要涉及一些与癌症相关的信号通路，如PI3K-Akt、p53、VEGF、Hippo通路。共表达分析结果表明有32个lncRNAs和81个mRNAs相互调节，其中起关键作用的是COL1A1、COL27A1、BMPR2等。作者接着分析了这些lncRNAs和mRNAs与肺癌患者预后的相关性，发现7个mRNAs和8个lncRNAs与患者的生存相关。2对lncRNA-mRNA与耐药高度相关。其中lncRNA（NR_028502.1/NR_028505.1）-MIR22HG（MIR22 host gene）与9个mRNAs共表达，可能在耐药中发挥抵抗作用，与肺癌患者更好的临床预后有关。lncRNA（NR_030725.1/NR_030726.1）-RCC1（regulator of chromosome condensation 1）与11个mRNAs共表达，可能促进耐药，与肺癌患者生存率差有关。这项研究的不足之处在于没有排除lncRNAs所具有的其他功能如染色质重塑、启动子甲基化、microRNA沉默的可能性等。

Wu等^[43]^分析了对gefitinib耐药的HCC827-8-1和亲本细胞HCC827的lncRNA表达谱，发现有1, 476个lncRNAs（703个上调、773个下调）和1, 026个mRNAs（516个上调、510个下调）表达失调。失调的mRNAs主要负责调节细胞外空间和受体结合等，涉及到的KEGG pathways主要与移植物抗宿主病、Ⅰ型糖尿病、病毒性心肌炎等有关。作者通过分析与lncRNAs共表达的mRNAs预测表达失调的lncRNAs的作用。结果表明这些lncRNAs主要在代谢、乙醛酸和二羧酸代谢、N-聚糖生物合成等信号途径中发挥作用。这项研究的结果为深入研究EGFR-TKIs的耐药机制提供了很多启示，作者在构建的lncRNA-TF（transcription factors）网络中，筛选出了4个关键基因：*E2F4*、*E2F1*、*uSF1*、*TFAP2C*。它们可能在EGFR-TKIs耐药中发挥重要作用，值得继续关注。

### 3.2 实验研究lncRNAs在NSCLC EGFR-TKIs耐药中的作用

迄今为止报道的lncRNAs与NSCLC耐药的研究多聚焦于顺铂等化疗药物的耐药^[2, 3, 28, 44, 45]^。有关lncRNAs在EGFR-TKIs耐药中的作用的研究报道尚不多见。

LncRNA-UCA1（urothelial cancer-associated 1）与细胞凋亡、增殖、化疗耐药相关^[39]^。Cheng等^[39, 46]^研究了NSCLC中lncRNA UCA1在EGFR-TKIs获得性耐药中的作用。结果表明，gefitinib耐药的PC9/R和H1975细胞过表达UCA1。相应的gefitinib获得性耐药的NSCLC患者UCA1 mRNA表达显著增加并且与患者的不良预后相关。进一步的分析发现这种相关性仅存在于没有T790M突变的NSCLC患者中。作者进一步通过体内外实验验证了这一发现。Western blot和免疫组化实验结果表明，UCA1与pEGFR、pAKT、pERK、pmTOR表达正相关。沉默UCA1表达使EMT相关的E-cadherin表达增强，vimentin、Snail、N-cadherin表达减弱。因此，UCA1可能通过活化AKT/mTOR和ERK通路和EMT促进gefitinib耐药。

已报道lncRNA BC087858在gefitinib耐药的NSCLC细胞中高表达^[39]^。Pan等^[47]^检测了不同NSCLC细胞和EGFR-TKIs耐药的肿瘤组织中lncRNA BC087858的表达，发现高表达lncRNA BC087858的NSCLC患者预后差。值得注意的是，这种显著的相关性也主要发生在T790M突变阴性的NSCLC患者中。体外siRNA-BC087858转染能够逆转T790M突变阴性的NSCLC耐药细胞对gefitinib的抗性并促进细胞凋亡和迁移。机制研究的结果表明，BC087858能够通过上调Snail和ZEB1表达促进EMT，也能通过上调pEGFR、pAKT、pERK表达活化PI3K/AKT和MEK/ERK信号通路，从而促进EGFR-TKIs耐药。

Wang等^[48]^分析了对gefitinib耐药的PC9-R细胞和亲本细胞的lncRNA表达谱之间的差异。结果表明PVT1、H19、MIR31HG、BOK-AS1、CBR3-AS1、LincRNA-p21差异表达。尤其是PVT1、MIR31HG、LincRNA-p21在耐药细胞中显著上调。转染siRNA-MIR31HG的PC9-R细胞对gefitinib的敏感性恢复。细胞凋亡率显著增加。细胞周期阻滞在G_0_期/G_1_期。Western blot结果表明，MIR31HG通过促进p-EGFR、p-PI3K、p-AKT、p-Mdm-2表达活化EGFR/PI3K/AKT信号通路在gefitinib耐药中发挥作用。

一个已知的LncRNA，GAS5（growth arrest-specific transcript 5）被报道在肺癌组织中表达降低^[49]^，并与较大的肿瘤体积、低分化、较高的TNM分期相关。Dong等^[50]^报道过表达GAS5能够逆转A549细胞对gefitinib耐药，这主要是由于过表达GAS5使p-EGFR、p-ERK、p-Akt、p-IGF-IR（insulin-like growth factor 1 receptor）的表达显著降低。本研究的一个亮点是作者通过体内实验验证了体外实验的结果，这对于后续的研究具有重要的指导意义。有报道^[51]^一个lncRNA SNHG12（small nucleolar RNA host gene 12）在NSCLC多药耐药中发挥作用。作者发现SNHG12在NSCLC细胞系和肿瘤组织中高表达。沉默SNHG12可通过诱导细胞凋亡逆转NSCLC细胞对cisplatin、paclitaxel、gefitinib的耐药。进一步的研究表明，SNHG12沉默通过上调miR-181a抑制MAPK1和MAP2K1表达，导致MAPK/Slug通路被抑制。该研究的不足之处在于体内实验仅验证了SNHG12对cisplatin治疗的调节作用。最近Wu等^[52]^报道gefitinib耐药细胞中LINC00635-001高表达。沉默LINC00635-001并没有对耐药的HCC827-8-1细胞产生影响。但是lncRNA沉默和gefitinib处理具有协同作用，可通过抑制AKT活化恢复耐药细胞对gefitinib的敏感性。

上述对lncRNAs在EGFR-TKIs耐药中的作用的研究报道多采用筛选差异表达lncRNAs并构建lncRNA与mRNA或蛋白共表达网络等生物信息学分析方法来进行，后续相关的实验验证和机制研究的报道还比较少见。分析原因除了样本的获得有难度之外，lncRNAs的非保守性和功能的多样性也为未来的研究带来了巨大的挑战。Sponge途径是lncRNAs发挥作用的经典方式，通过双荧光报告实验即可初步阐明lncRNAs的作用机制，但是ceRNA调控必须在对应的miRNA和lncRNA达到一定丰度时才能发挥作用。因此需要通过Northern blot和FISH等实验证明所研究的miRNA和lncRNA的高表达和胞浆分布，这无疑会使很多候选的lncRNAs被淘汰。另外，大多数的lncRNAs都分布于核中，因此lncRNAs对基因和蛋白表达的调控才是其主要的作用方式。这就需要进行RNA pull down、质谱检测等有难度的验证实验。相信随着生物信息学等学科的飞速发展，lncRNAs相关的算法和数据库会更加完善，针对lncRNAs的预测结果也会更加准确，从而可以使未来的研究进程加速进行^[53]^。

## 4 问题与展望

在目前的临床治疗中NSCLC EGFR-TKIs耐药仍是需要重点解决的问题。因此探究EGFR-TKIs的耐药机制，寻找有效的克服耐药的手段至关重要。非编码RNA的发现为研究EGFR-TKIs的耐药提供了新的思路，同时也意味着更复杂的耐药机制需要被阐明。lncRNAs是长度大于200 nt的非编码RNA，在基因表达调控中发挥基础性作用。随着越来越多的研究发现lncRNAs广泛参与多种肿瘤的发生发展进程，它们有望作为一类极有前途的组织和/或血液系统的肿瘤标志物在临床治疗中发挥作用。本文综述了lncRNAs在NSCLC患者EGFR-TKIs耐药中的作用。这些lncRNAs既可以作为耐药的标志，也可以作为诊断和治疗的新靶点。目前最重要的问题是需要对它们进行详细的研究并早日在临床应用。众所周知，绝大多数lncRNAs的作用机制和生物学功能现在都是未知的，因此未来的工作面临巨大的挑战。要早日实现这样的目标需要基础研究工作者和临床医生通力合作并综合利用多种新技术新方法，例如建立精确调节lncRNAs表达的实验体系、获得足够的研究样本等。可喜的是，最近生物信息学的迅猛发展已经大大加速了lncRNAs的研究进程。其他新近发展的技术如CRISPR-Cas9系统等也可以帮助我们进行有效靶向基因组的实验设计。期待在不远的将来有更多的肿瘤患者能够在精准治疗中获得更好的临床疗效。
